# Advances in biomarker discovery for canine cognitive dysfunction: a comprehensive structured narrative review and future perspectives

**DOI:** 10.1007/s11259-026-11425-8

**Published:** 2026-08-07

**Authors:** Klysse Assumpção Barbosa, Heloísa Máximo Ribeiro, Luíz Guilherme Dercore Benevenuto, Paulo César Maiorka, Marcia Regina Cominetti, Rogério Martins Amorim, Didier Quevedo Cagnini

**Affiliations:** 1 Department of Veterinary Clinic, School of Veterinary Medicine and Animal Science, São Paulo State University - UNESP, Botucatu, SP Brazil; 2 Department of Gerontology, Federal University of São Carlos, São Carlos, SP Brazil; 3 Department of Pathology, Faculty of Veterinary Medicine and Animal Sciences, University of Sao Paulo, Sao Paulo, Brazil; 4 Department of Veterinary Surgery and Anesthesiology, School of Veterinary Medicine and Animal Science, São Paulo State University - UNESP, Botucatu, São Paulo Brazil

**Keywords:** Amyloid-β, Biomarkers, Canines, Canine cognitive dysfunction, Dogs, Glial fibrillary acidic protein, Neurofilament-light chain, Tau, Translational medicine

## Abstract

Canine Cognitive Dysfunction (CCD) is a naturally occurring neurodegenerative syndrome in aging dogs that shares clinical and neuropathological parallels with Alzheimer’s disease (AD). As the demand for objective diagnostic tools grows, identifying reliable biofluid biomarkers is essential for clinical staging and therapeutic monitoring. This review synthesizes evidence on cerebrospinal fluid (CSF) and blood-based biomarkers (BBM) of CCD, focusing on amyloid-β (Aβ), neurofilament light chain (NfL), tau, and glial fibrillary acidic protein (GFAP). Evidence shows that Aβ42 and Aβ42/Aβ40 ratios exhibit stage-dependent, non-linear alterations resembling early compensatory phases in human AD. In contrast, tau pathology in CCD consists mainly of pre-tangle synaptic hyperphosphorylation rather than abundant neurofibrillary tangles, limiting its current diagnostic utility. GFAP, a marker of astroglial activation, shows inconsistent associations with cognitive decline and remains exploratory. Conversely, NfL has emerged as the most robust biomarker; CSF and plasma NfL levels consistently increase with age, correlate with cognitive impairment, and reflect central axonal pathology, making it the leading candidate for staging and monitoring disease progression. Overall, the CCD biomarker landscape supports a multimodal approach integrating Aβ dysregulation, axonal injury, and glial activation. Advancing this field requires harmonized diagnostic criteria, standardized sampling, and longitudinal studies. Such efforts will strengthen the translational value of CCD as a model for human dementia, accelerating discovery and therapeutic development across species.

## Background

In recent decades, veterinary and human medicine have witnessed a remarkable rise in life expectancy, driven by advances in healthcare access, nutrition, diagnostics, and therapeutics (Leulier et al. 2017). While this progress has greatly improved the quality of life, it has also led to an increased prevalence of chronic, progressive neurodegenerative disorders in humans and companion animals (Van Schependom and D’Haeseleer 2023). Among companion animals, canine cognitive dysfunction (CCD) is one of the most common age-related neurological conditions (Chapagain et al. 2018).

CCD exhibits several clinically and biologically relevant parallels to Alzheimer’s disease (AD), not only in the clinical signs, but also in neuropathological features. Clinical manifestations of CCD are commonly summarized by the DISHAA acronym and include disorientation, altered social interaction, sleep-wake cycle disturbances, house soiling, changes in activity levels, and anxiety (Landsberg et al. 2012). Pathological hallmarks include β-amyloid (Aβ) deposition in the brain parenchyma and vasculature, tau hyperphosphorylation and aggregation, synaptic loss, and progressive cognitive impairment (Ruehl et al. 1995). Importantly, dogs share similar neuroanatomy, comparable environmental exposures, and lifestyle influences with their human caregivers. These characteristics make the aging dog a highly relevant spontaneous translational model for elucidating mechanisms that drive human dementia and for evaluating novel diagnostic and therapeutic strategies (Devinsky et al. 2018).

Epidemiological data indicate substantial variation in CCD prevalence, with estimates ranging from ~ 14% to ~ 65% among older dogs and increasing sharply with age (Figueroa et al. 2025; Salvin et al. 2011). This wide range likely reflects differences in study design, populations, and diagnostic approaches, as clinical diagnosis currently relies on behavioral history, neurological examination, and the application of validated instruments such as the Canine Dementia Scale (CADES) (Madari et al. 2015) and the Canine Cognitive Dysfunction Rating Scale (CCDR) (Salvin et al. 2011). Complementary assessments, including routine laboratory testing, cerebrospinal fluid (CSF) analysis, and magnetic resonance imaging, help exclude other causes of cognitive decline and detect age-related neuroanatomical alterations (Ehrenzweig and Hunter 2023).

However, clinical diagnosis is often established at moderate to advanced stages when behavioral symptoms are evident and therapeutic options are less effective. This limitation has accelerated the search for objective, minimally invasive biomarkers that support early detection, monitor disease progression, and improve therapeutic decision-making (Ribeiro et al. 2026).

Historically, biomarker efforts in CCD, as in human AD, have centered on Aβ-related pathology (Hines et al. 2023). Yet, growing evidence underscores that CCD is a multifactorial disorder, and that biomarkers capturing downstream neurodegeneration and glial responses may provide complementary and clinically additional information. Investigations have explored neurofilament light chain (NfL) (Alsulami et al. 2025; Sung et al. 2024), tau abnormalities (Abey et al. 2021), and glial fibrillary protein (GFAP) (Alsulami et al. 2025; Yoon et al. 2025). Together, these markers may provide a more comprehensive understanding of neurodegeneration in aging dogs.

Therefore, this structured narrative review synthesizes the knowledge on Aβ-, tau-, NfL-, and GFAP-related biomarkers for CCD, discussing their biological significance, diagnostic performance, and translational implications for human neurodegenerative research. By identifying key advances and gaps, we aim to support the development of earlier, more precise diagnostic strategies that ultimately benefit both canine and human brain health.

## Main text

### Materials and methods

For this structured narrative review, searches focused on studies evaluating biomarkers associated with CCD were conducted in PubMed, Web of Science, and Scopus through September 2025 using combinations of the terms “canine cognitive dysfunction”, “dog”, “biomarker”, “amyloid”, “neurofilament light”, “tau”, and “GFAP”. Eligible records were restricted to original research articles reporting primary biomarker data from canine samples. Reference lists of included articles were manually screened to identify additional relevant studies.

Eligibility criteria were applied to enhance transparency and methodological rigor. Studies were included if they: (i) involved dogs evaluated for aging and/or cognitive decline, and (ii) reported biomarker measurements in brain tissue, CSF, or blood relevant to amyloid-β (Aβ), NfL, tau, and/or GFAP. Reports exclusively involving non-canine species, single-case descriptions, or therapeutic interventions were not included. Reviews, case reports, and conference abstracts were used only for background and interpretative support.

Data were narratively summarized, with emphasis on sample characteristics (breed composition, age range, and cognitive phenotyping), analytical platforms (e.g., enzyme-linked immunosorbent assay - ELISA, single-molecule array - SIMOA, mass spectrometry), and biomarker outcomes, including their reported associations with cognitive status. Given the heterogeneity in study design, methods, and outcome measures across studies, a qualitative synthesis was prioritized over meta-analytic pooling to identify convergent evidence, inconsistencies, and knowledge gaps.

This work is classified as a structured narrative review, a methodology that combines a systematic and reproducible search strategy with qualitative, narrative synthesis. This approach differs from a formal systematic review (e.g., PRISMA-adherent) in that it does not mandate exhaustive retrieval of all eligible studies, nor does it perform quantitative data pooling. Instead, it employs defined search terms, explicit database coverage, and eligibility criteria to ensure comprehensive and transparent literature retrieval, while retaining the interpretive flexibility required to synthesize a heterogeneous body of evidence spanning diverse study designs, species-specific contexts, and analytical platforms. This classification is consistent with established frameworks for structured narrative reviews in biomedical research (Green et al. 2006; Ferrari 2015).

### Overview

**Fig. 1:**
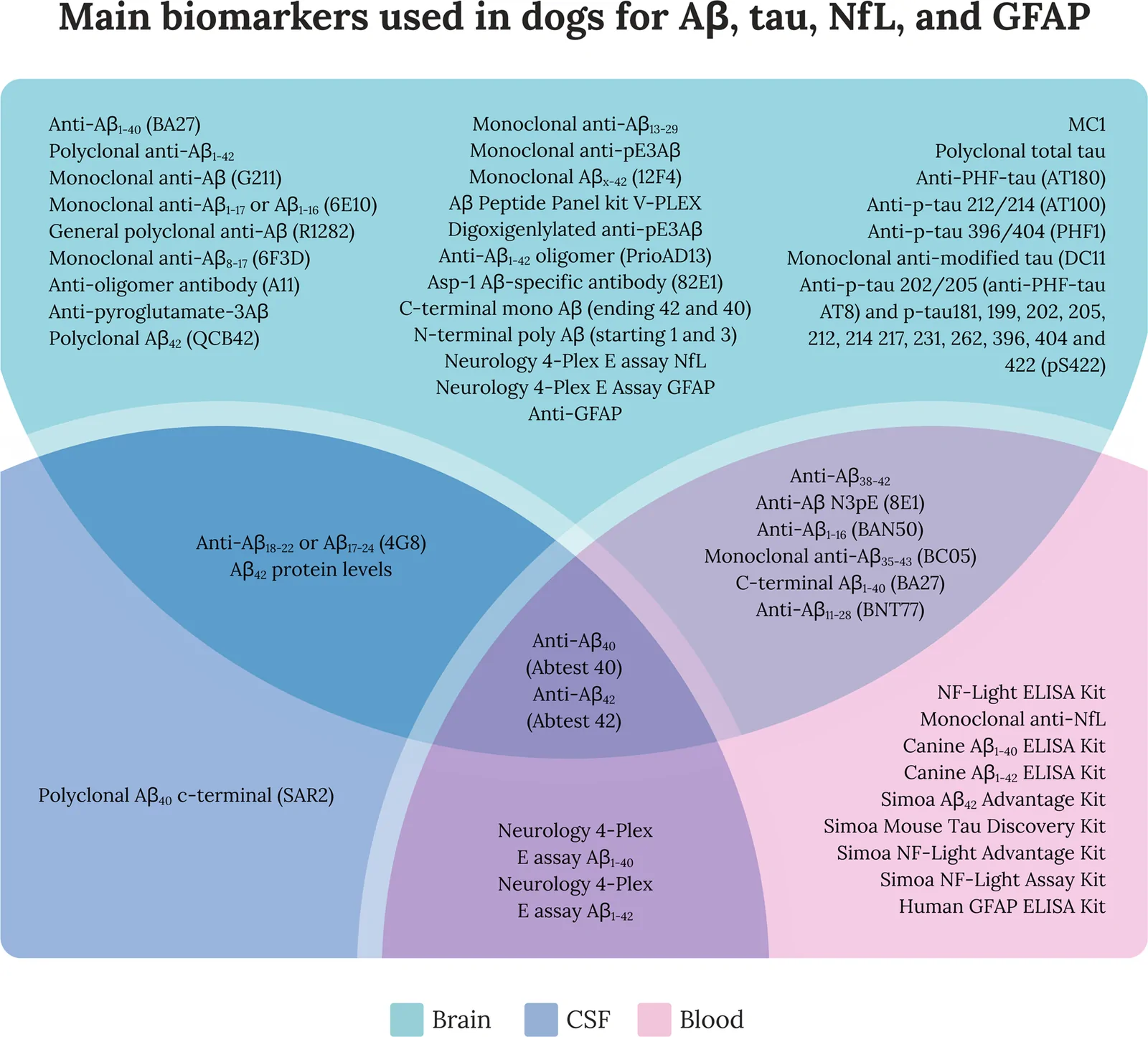
Adapted Venn diagram showing the main biomarkers retrieved from the review for Aβ, tau, NfL, and GFAP. Biomarkers used in brain tissue (light green), in CSF (blue), and in blood (pink). The intermediate colors at the intersection points show the biomarkers used in brain and CSF, brain and blood, blood and brain, or in all three types of samples at the center of the diagram. The main techniques where biomarkers were applied were immunohistochemistry, ELISA, and Single Molecule Array (SIMOA – Quanterix)

**Fig. 2:**
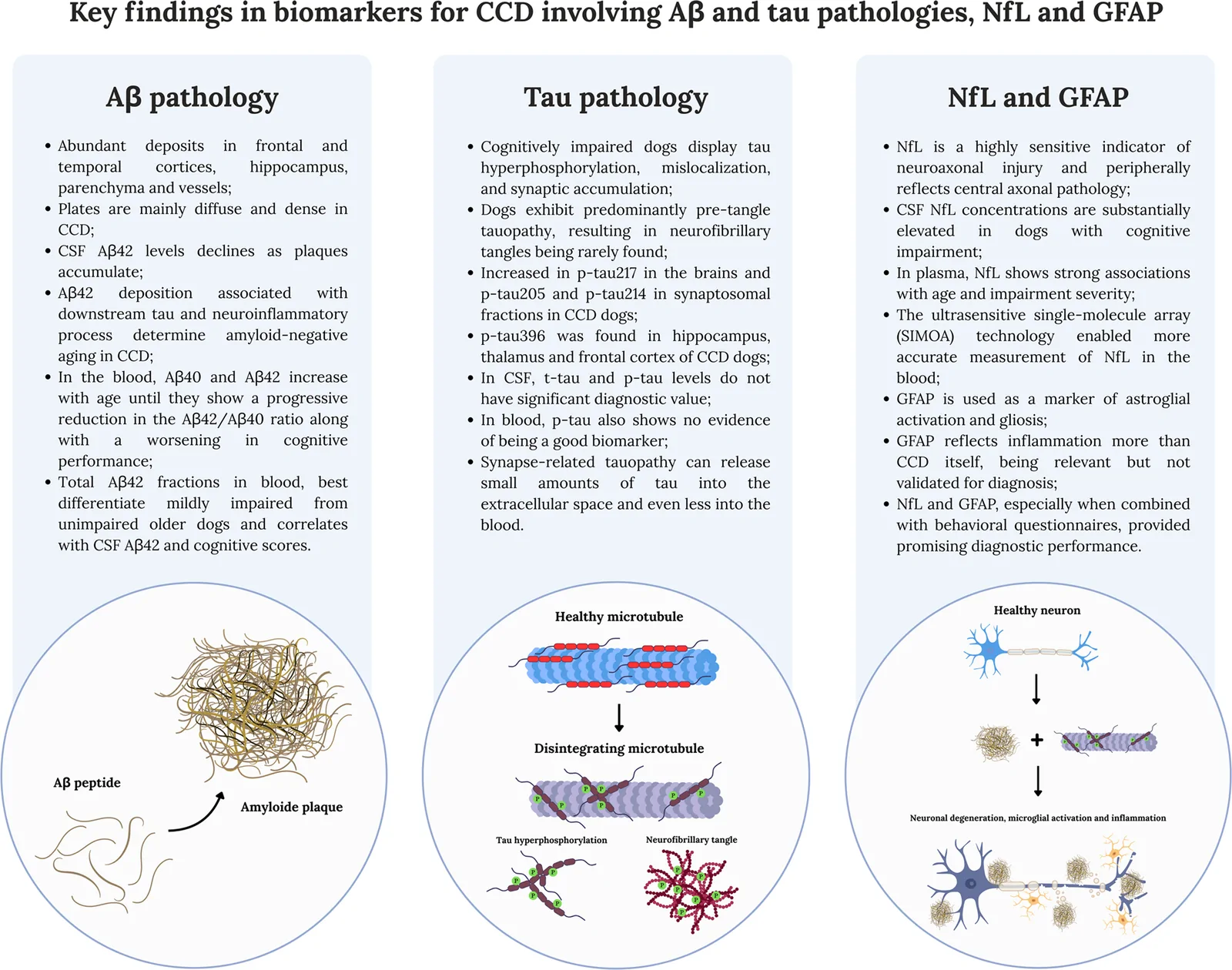
Summary of the main outcomes and conclusions on Aβ and tau pathologies, NfL, and GFAP highlighted in the review, together with the respective schematic representations of the key pathophysiological changes. Aβ pathology: in CCD, the amyloidogenic cleavage pathway of APP is predominant, leading to the buildup of Aβ peptides that accumulate in Aβ plaques. Tau pathology: hyperphosphorylation of tau protein leads to destabilization of microtubules, which rarely agglomerate into neurofibrillary tangles (NFTs) in dogs. NfL and GFAP: microtubule destabilization combined with the accumulation of Aβ plaques and NFTs leads to inflammation and degeneration of brain tissue, causing loss/reduction of its function

The main biomarkers found for each type of sample analyzed, such as brain, cerebrospinal fluid, and blood, separately and together, are summarized in Fig. 1. Meanwhile, Fig. 2 shows the results discussed throughout the article regarding Aβ and Tau pathologies, (NfL), and GFAP, along with a schematic representation of their pathophysiologies. The following sections present discussions of the articles, organized by the same division: papers on Aβ, Tau, NfL, and GFAP, focusing on analyses of brain tissue, CSF, and blood (plasma or serum).

### Amyloid-β (Aβ)

The beta-amyloid (Aβ) peptide is a critical proteolytic derivative of the transmembrane Amyloid Precursor Protein (APP), generated through sequential cleavage by β-secretase and γ-secretase enzymes (Brothers et al. 2018; Hardy and Higgins 1992). Under normal physiological conditions, soluble Aβ monomers contribute to neuroprotective functions, including antimicrobial defense and the modulation of synaptic plasticity and excitatory homeostasis (Brothers et al. 2018). However, a pathological shift occurs when a stoichiometric imbalance leads to the accumulation of the Aβ_42_ isoform, which possesses a high propensity for misfolding into toxic oligomers (Selkoe and Hardy 2016).

Before the late 1980s, antibodies specific to Aβ were not yet widely available, making earlier descriptions of “senile” or “neuritic” plaques difficult to interpret within modern neuropathological terminology. One of the first systematic examinations of Aβ in the canine brain evaluated more than 100 dogs using immunohistochemistry, modified Bielschowsky silver staining, thioflavin S, and Congo red (Cummings et al. 1996). The authors observed Aβ deposition and immunoreactive amyloid plaques in over half of the animals aged 10 years or older. However, dense-core, fibrillar plaques were uncommon, as Congo red-positive or thioflavin S-positive lesions were not consistently detected even in dogs aged 17–18 years. This lack of congophilia persisted despite fluorescence-based detection methods and ultraviolet excitation for thioflavin S, which are more sensitive than traditional polarized light microscopy. Collectively, these early findings suggest that although aged dogs accumulate substantial Aβ, plaques often remain diffuse and may not mature into the highly fibrillar, congophilic structures typical of advanced human AD.

Over subsequent decades, research on canine Aβ accumulation has substantially deepened our understanding of CCD as a naturally occurring model of AD-type amyloidosis. The key studies are summarized in Table 1. One influential study by Head et al. (2010) quantified Aβ40, Aβ42, and oligomeric assemblies in the prefrontal cortex and CSF of aged beagles using target-specific antibodies. For instance, conjugated BA27 (for Aβ40) or BCO5 (for Aβ42) were used to demonstrate that SDS-soluble Aβ42 and Aβ oligomers correlated strongly with age and cognitive deficits. For clarity and consistency, the antibodies are referenced here by clone/target: BA27 (anti-Aβ40) and BC05 (anti-Aβ42/43). Using these reagents, the authors reported that SDS-soluble Aβ42 and Aβ oligomers increased with age and correlated with cognitive deficits, whereas total plaque burden alone did not fully predict impairment (Head et al. 2010). This work supported the concept that soluble Aβ species may be more strongly associated with cognitive outcomes than plaque load, paralleling observations in human AD. Accordingly, as aging dogs accumulate Aβ in vulnerable cortical regions, CSF Aβ42 tends to decline in association with increasing amyloid burden, consistent with the inverse relationship widely reported in AD (Fagan et al. 2006; Head et al. 2010).

**Table 1:** Summary of brain, cerebrospinal fluid (CSF), and blood biomarkers investigated in canine cognitive dysfunction (CCD)*

Study	Breed	Age	Total (*n*)	**Sample**	**Assay**	Antibodies	Main findings
**A | Amyloid Beta (Aβ) **							
Torp et al. (2000)	Beagles and mixed breed	1 to 12	8	Brain	IHC	Polyclonal anti-Aβ1–42 and monoclonal anti-Aβ (G211)	In aged dogs, Aβ accumulates as membrane-associated fibrillar deposits that increase with age, localize to neuronal and vascular membranes, and are linked to progressive ultrastructural damage, including demyelination and neuronal degeneration.
Head et al. (2010)	Beagles	4 and 12	2	Brain	IHC	Monoclonal anti-Aβ1–16 (6E10)	The aged canine brain exhibited diffuse plaque formation, particularly in deep cortical layers in the hippocampus and entorhinal and frontal cortices.
Miyawaki et al. (2002)	Shih-tzu, Maltese, Shetland sheepdog and Dalmatian	10 to 17	5	Brain	IHC	Monoclonal anti-Aβ35–43 (BC05) and anti-Aβ1–40 (BA27)	In aged dogs, diffuse amyloid plaques are composed predominantly of Aβ42(43) with a granular, non-fibrillar morphology, whereas mature plaques contain Aβ40 alone or in combination with Aβ42(43), indicating that diffuse and mature plaques arise through distinct pathogenic processes rather than a linear progression.
Tapp et al. (2004)	Beagles	2 to 15	23	Brain	IHC	Polyclonal anti-Aβ1–42	Aβ deposition in the frontal cortex increased with age, and higher Aβ42 immunolabeling was strongly associated with smaller frontal lobe volumes. The degree of frontal Aβ accumulation also correlated with poorer performance on executive-function tasks.
Pugliese et al. (2006)	Various	1 to 20	10	Brain	IHC	Monoclonal anti-Aβ8–17	Widespread diffuse Aβ plaques are found throughout the cerebral cortex. Its density increased with age and with the severity of cognitive deficits.
Bernedo et al. (2009)	Various	< 7 to > 10	19	Brain	IHC	Monoclonal anti-Aβ4–9 (Aβ42, 6E10) and anti-Aβ18–22 (Aβ42, 4G8)	In aged dogs with cognitive impairment, there are extensive diffuse cortical Aβ deposits. Young dogs and most aged cognitively normal dogs showed little to no Aβ accumulation.
Nuntagij et al. (2009)	Not reported	Aged	Not reported	Brain	IHC	Polyclonal anti-Aβ1–42 and monoclonal anti-Aβ1–17 (6E10)	In aged dogs, early amyloid-β deposition occurs as membrane-associated, thread-like fibrils closely apposed to neuronal dendrites and cell bodies, indicating that amyloid fibrillogenesis is a localized, membrane-driven process rather than random extracellular plaque formation.
Head et al. (2010)	Beagles	4.5 to 15.7	30	Brain	IHC	Polyclonal anti-Aβ1–42	In aged canines, Aβ42 accumulated earlier and more prominently than Aβ40 in the prefrontal cortex, with an age-dependent exponential rise in cortical Aβ42 load. In CSF, Aβ42 (but not Aβ40) declined with age, and lower CSF Aβ42 percentages strongly predicted higher brain Aβ42 and Aβ40 levels, establishing a direct inverse relationship between CSF Aβ42 and cortical amyloid burden in dogs.
					ELISA	Anti-Aβ42 (BC05) and anti-Aβ40 (BA27)	
					WB	Anti-oligomer antibody (A11)	
González-Martínez et al. (2011)	Not reported	1 to > 9	88	Plasma	ELISA	Anti-Aβ1 − 40 (ABtest 40) and anti-Aβ1 − 42 (ABtest 42)	Plasma Aβ42 and Aβ40 decline with normal aging but rise transiently in mild cognitive impairment, showing the highest Aβ42 levels and Aβ42/40 ratios, before decreasing again in severe impairment.
Chambers et al. (2011)	Various	1 to 20	47	Brain	IHC	C-terminal: monoclonal Aβ ending at 42 and 40. N-terminal: polyclonal Aβ starting at 1 and 3.	Amyloid deposition shifts with age from N-terminally intact Aβ to pyroglutamate AβpN3, which accumulates in an age-dependent manner and becomes the dominant component of senile plaques and vascular amyloid in very old animals.
Pop et al. (2012)	Beagles	1 to 14.25	21	Brain	IHC	Monoclonal anti-Aβ1–17 (6E10)	In aging dogs, both soluble and insoluble Aβ40 and Aβ42 increase across cortical regions, with Aβ42 rising earlier and more prominently, alongside accumulation of toxic oligomeric species, reflecting an age-related shift toward amyloidogenic APP processing that promotes Aβ production and aggregation.
					ELISA	Monoclonal anti-Aβ1–16 and anti-aβx40 or anti-aβx42	
Fast et al. (2013)	Various	5 to 17	20	Brain	IHC	Monoclonal anti-Aβ1–16 (6E10)	In cognitively impaired aged dogs, diffuse Aβ and AβPP accumulate widely in cortical and hippocampal regions without forming fibrillar plaques, and these deposits do not bind [¹¹C]PiB, highlighting fundamental differences between canine and human amyloid pathology.
Frost et al. (2013)	Beagles	10 to 14	3	Brain	IHC	General polyclonal anti-Aβ (R1282); monoclonal anti-Aβ1–16 (3–8, 6E10); anti-pyroglutamate-3 Aβ; polyclonal Aβ42 (QCB42); Asp-1 Aβ-specific antibody (82E1)	In aged beagle dogs, pE3-Aβ was detected in cortical plaques and, even more prominently, in cerebral blood vessels, where deposition began around 12–14 years of age; pE3-Aβ colocalized with general Aβ42 staining but was present in only a subset of plaques, with vascular amyloid showing the strongest immunoreactivity.
Sarasa et al. (2013)	Not reported	Not reported	Not reported	CSF	IP-MALDI	Monoclonal anti-Aβ1–16 (4–9, 6E10); Monoclonal anti-Aβ18–22 (4G8); polyclonal Aβ40 C-terminal (SAR2)	Canine CSF contains a wide repertoire of Aβ species nearly identical to those found in human CSF, including Aβ1–33, Aβ1–34, Aβ1–37, Aβ1–38, Aβ1–39, Aβ1–40, and Aβ1–42, along with numerous N-terminally truncated peptides such as Aβ3–40 and Aβ11–40, confirming that dogs generate Aβ through the same β- and γ-secretase pathways as humans. Importantly, three low-molecular-weight species present in human CSF (Aβ1–13, Aβ1–14, Aβ1–18) were absent in dogs, but the overall similarity of Aβ profiles strongly supports the dog as a biologically relevant model for studying early amyloidogenic processes in Alzheimer-related research.
Schütt et al. (2015)	Various	8 to 15	51	Plasma; Brain	ELISA	C-terminal: anti- Aβ1–40 (BA27) and anti Aβ35–43 (BC05); N-terminal: anti-Aβ1–16 (BAN50) and anti-Aβ11–28 (BNT77). Pyroglutamate-modified Aβ: Aβ38–42 and anti-Aβ N3pE (8E1)	Plasma Aβ42 levels were significantly higher in dogs with CCD than in both mildly impaired and cognitively normal dogs. Aβ42 concentrations showed a positive correlation with CCDR scores.
Schmidt et al. (2015)	Various	2 and 8 to 19	24	Brain	IHC; IF	Aβ8–17 (clone 6F3D); anti-Aβ13–29; monoclonal anti-pE3Aβ (clone 2–48); digoxigenylated anti-pE3Aβ.	In dogs, amyloid deposition emerges around 9–11 years of age and progresses regionally from diffuse to dense-core plaques, with pyroglutamate-Aβ becoming the predominant species and increasing strongly with age, particularly in smaller breeds, alongside local astrocytic and microglial activation.
Ozawa et al. (2016)	Various	5 to 18	37	Brain	IHC	Monoclonal Aβx-42 (clone 12F4)	In dogs, diffuse Aβ42 senile plaques emerge around 10 years, peak in mid-old age, and decline in the oldest animals, whereas Aβ42-positive cerebral amyloid angiopathy increases steadily with age; neither plaque nor CAA burden correlates with cognitive impairment, indicating that amyloid deposition alone does not drive CCD.
Smolek et al. (2016)	Various	1 to 19	28	Brain	IHC	Anti-Aβ17–24 (4G8)	The frontal and temporal cortices were the most affected by AB pathology. Three types of amyloid deposits were found: condensed, diffuse cloud-like, and perivascular plaques. Plaques were not detected in white matter, and dogs without dementia did not show condensed plaques or vascular amyloid deposition.
Vikartovska et al. (2021)	Various	< 3 and > 8	39	Brain	IHC	Monoclonal anti-Aβ18–22 (4G8)	No tau pathology or AT8-positive neurofibrillary tangles were detected in the frontal cortex or hippocampus of dogs with mild cognitive impairment and those with severe CCD, despite abundant Aβ plaques and microglial activation.
				Serum	Quanterix Simoa	Simoa Aβ-42 Advantage Kit.	
Stylianaki et al. (2020)	Various	0 to 17	61	CSF and Plasma	ELISA	Aβtest40 and Aβtest42	In dogs, early cognitive impairment is associated with reduced CSF Aβ42 and stable CSF Aβ40, alongside a transient increase in plasma Aβ42, Aβ42/Aβ40, and plasma-bound Aβ42, indicating a non-linear, stage-dependent amyloid pattern consistent with early AD-like changes.
Habiba et al. (2021)	Papillon; Pomeranian; Lhasa Apso; Shiba Inu; Mongrels	14 to 17	7	Brain	IHC	Monoclonal anti-Aβp (4G8)	In CCD dogs, diffuse amyloid plaques and cerebral amyloid angiopathy coexist with marked intraneuronal Aβ1–42 oligomer accumulation in hippocampal and frontal regions, which colocalizes with hyperphosphorylated tau
					IF	Monoclonal anti-Aβp (4G8) and anti-Aβ1–42 oligomer (PrioAD13)	
Urfer et al. (2021)	Various	1 to 18	45	CSF and Brain	Luminex	Aβ42 protein levels	Aβ42 levels in brain tissue increase with age across different brain regions. Higher brain Aβ42 concentrations correlated with worse cognitive scores, even after adjusting for age in most regions. CSF Aβ42 declined with age and was unrelated to cognition.
Hines et al. (2023)	Various	0.9 to 16	47	Brain	IHC	Polyclonal anti-Aβ1–42	Intracellular Aβ1–42 accumulation increases with age and lower cognition. Intracellular Aβ pathology, not plaque formation, may be a relevant correlate of CCD in naturally aging dogs.
Alsulami et al. (2025)	Various	2 to 16.7	54	Brain	MSD	Aβ40 and Aβ42 (Aβ Peptide Panel kit V-PLEX)	Aβ1–42 levels in the frontal cortex were significantly higher in aged dogs compared to young dogs. CSF Aβ1–42/Aβ1–40 ratio was significantly lower in CCD+ dogs and correlated negatively with CADES scores.
				Plasma and CSF	Quanterix Simoa	Neurology 4-Plex E assay Aβ1–40 and Aβ1–42	
Yoon et al. (2025)	Various	1.3 to 17.42	77	Plasma	ELISA	Canine Aβ1–40 and 1–42 ELISA Kits	Plasma Aβ40 and Aβ42 concentrations did not differ significantly between cognitively normal dogs, dogs with mild cognitive impairment, and those with CCD, and neither peptide showed meaningful correlations with cognitive test performance or clinical scores.
**B | Tau**							
Pugliese et al. (2006)	Various	1 to 20	10	Brain	IHC	Polyclonal anti-ptau181, p-tau202 and p-tau396	No spatial relationship between Aβ plaques and tau hyperphosphorylation.
Schmidt et al. (2015)	Various	8 to 19	24	Brain	IHC; IF	Anti-ptau202/205 (AT8); ptau212/214 (AT100); MC1 (detects abnormalfolded tau); p-tau396/404 (PHF1); ptau422 (pS422)	In aged dogs, tau pathology was detected in only a small subset of animals, showing severe neuronal degeneration but no correlation with amyloid burden, suggesting that tau accumulation is an infrequent, age-related process independent of Aβ deposition.
Smolek et al. (2016)	Various	1 to 19	28	Brain	IHC	Anti-ptau199; 205;212, 214; 217; 262,396; 404; monoclonalantimodified-tau(DC11); Anti-PHF-tau (AT8 andAT180); polyclonaltotal tau	Aged dogs with cognitive impairment exhibited significant tau hyperphosphorylation within hippocampal synaptosomes, indicating that tau pathology in these animals is enriched at synaptic terminals rather than forming classical neurofibrillary tangles. This synaptic tau hyperphosphorylation correlated with markers of neuroinflammation.
Vikartovska et al. (2021)	Various	<3 and >8	39	Brain	IHC	Anti-PHF-ptau202/205 (AT8)	No tau pathology or AT8-positive neurofibrillary tangles were detected in the brain of dogs with mild cognitive impairment and those with severe CCD, despite abundant Aβ plaques and microglial activation.
				Serum	Quanterix Simoa	Simoa Mouse Tau Discovery Kit(Quanterix);	
Abey et al.(2021)	Various	14 to 17	12	Brain	EnVision IHC	Anti-PHFTau(AT8)	In aged dogs with CCD, p-tau396 was elevated across multiple brain regions. By contrast, AT8-positive neurofibrillary tangles were rare, indicating that CCD exhibits early-stage tauopathy.
					EnVision IHC and IF	Anti-ptau396	
					WB	Anti-ptau396	
Habiba et al. (2021)	Papillon;Pomeranian;Lhasa Apso;Shiba Inu;Mongrels	14 to 17	7	Brain	IHC; IF	Anti-ptau202/205 (AT8)	In aged dogs with CCD, AT8-positive p-tau was widespread throughoutneurons. P-tau strongly co-localized with intracellular Aβ1-42 oligomers, but not with diffuse Aβ plaques, indicating a pathogenic interaction between tau dysregulation and oligomeric Aβ that may drive synaptic dysfunction and cognitive decline.
Hines et al.(2023)	Various	0.9 to 16	47	Brain	IHC	Anti-ptau181 and 217	In aging dogs, p-tau181 and 217 increased in the brain, with some animals showing intracellular aggregates and fibril-like structures. p-tau217 was found exclusively in dogs with CCD, suggesting that this phosphorylation site may represent a CCD-specific pathological marker.
Alsulami et al. (2025)	Various	2 to 16.7	54	Brain	IHC	Anti-ptau217 and231	Increased p-tau217 and 231 in the brain of CCD-positive dogs relative tocognitively unimpaired aged controls indicated enhanced tau pathologyassociated with cognitive decline. No significant group differences in ptau396, suggesting that tau abnormalities in CCD may be phosphorylationsite specific rather than globally elevated.
					MSD	Anti-ptau396	
**C | NfL**							
Panek et al.(2020)	Various	0.4 to 15.6	63	Plasma	Quanterix Simoa	Simoa NFLight Assay kit	Plasma NfL increases with age in healthy dogs. CCD and degenerativemyelopathy dogs had higher NfL levels than age-matched controls. In older dogs, plasma NfL correlated positively with CADES dementia scores.
Vikartovska et al. (2020)	Various	3 to 8	39	Serum	Quanterix Simoa	Simoa NFLightAdvantage kit	Serum NfL levels were elevated in dogs with mild cognitive impairment,compared to age-matched normally aging dogs and young controls. Serum NfL significantly correlated with CADES scores.
Perino et al.(2021)	LabradorRetrievers	1.4 to 14.8	55	Plasma	Quanterix Simoa	Simoa NFLightAdvantage Kit	Plasma NfL concentrations increased significantly with age in healthyLabrador Retrievers and lower body weight and shorter height wereindependently associated with higher plasma NfL levels, indicating thatmorphological factors influence baseline NfL values.
Fefer et al. (2022)	Various	9.3 to 15.3	39	Plasma	Quanterix Simoa	Simoa NFLight Assay kit	Plasma NfL was significantly higher in cognitively impaired dogs, and these elevated levels correlated strongly with both worse inhibitory controlperformance and higher CADES dementia scores. Among all biomarkers and behavioral measures tested, NfL showed the strongest association with cognitive decline, supporting its utility as a sensitive marker of neurodegeneration in aging pet dogs.
Wu et al.(2023)	Various	3 to 17	36	Plasma	IMR	Monoclonalanti-NfL	Plasma NfL was significantly higher in dogs with CCDS than in cognitivelynormal dogs, with IMR technology clearly separating CCDS from non-CCDS cases.
Alsulami etal. (2025)	Various	2 to 16.7	54	Brain	Quanterix	Neurology 4-Plex E assay NfL	Plasma and CSF concentrations of NfL were higher in older dogs with andwithout dementia than in younger dogs. Plasma concentrations were higher in dogs with CCD, but not in CSF.
Yoon et al.(2025)	Various	1.3 to 17.42	77	Serum	ELISA	NF-lightELISA Kit	Plasma NfL and GFAP were significantly elevated in dogs with CCD compared to cognitively normal and mildly impaired dogs, and showed strong associations with cognitive decline, with NfL demonstrating the highest diagnostic accuracy among all biomarkers tested.
**D | GFAP**							
Torp et al. (2000)	Beagles and mixed breed	1 to 12	8	Brain	IHC	Anti-GFAP	GFAP was intimately associated with AB fibers in areas of its extensive deposition, with disruption of the glial plasma membrane.
Schmidt et al. (2015)	Various	8 to 19	24	Brain	IHC; IF	Anti-GFAP	A slight increase in GFAP expression indicates astrocytic reactivity.
Ozawa et al. (2016)	Various	5 to 18	37	Brain	IHC	Anti-GFAP	Increased GFAP-positive astrocytes with activated shape in aged dog.
Abey et al. (2021)	Various	14 to 17	12	Brain	IF	Anti-GFAP	Glial nuclei associated with S396+ accumulations are not in microglia or astrocytes (no colocalization of S396 signal with GFAP).
Hines et al. (2023)	Various	0.9 to 16	47	Brain	IHC	Anti-GFAP	No significant increase in GFAP+ astrocytes was seen.
Alsulami et al. (2025)	Various	2 to 16.7	54	Brain	Quanterix	Neurology 4-Plex E assay GFAP	CSF and plasma NfL concentrations increased with age. Plasma GFPAP NfL correlated with CADES scores and with each other.
Yoon et al. (2025)	Various	1.3 to 17.42	77	Plasma	ELISA	Human GFAP ELISA Kit	Plasma GFAP increased progressively with disease severity.

Age range is presented in years

*Aβ* Amyloid-β, *Aβ40* Amyloid-β 1–40, *Aβ42* Amyloid-β 1–42, *AβpN3/pE3-Aβ* Pyroglutamate-3–modified amyloid-β, *AD* Alzheimer’s disease, *APP* Amyloid precursor; protein, *AT8* Antibody recognizing phosphorylated tau at Ser202/Thr205, *AT100* Antibody recognizing phosphorylated tau at Thr212/Ser214, *CADES* Canine Dementia Scale, *CCD* Canine cognitive dysfunction, *CCDR* Canine Cognitive Dysfunction Rating Scale, *CSF* Cerebrospinal fluid, *ELISA* Enzyme-linked immunosorbent assay, *GFAP* Glial fibrillary acidic protein, *IHC* Immunohistochemistry, *IF* Immunofluorescence, *IMR* Immunomagnetic reduction, *IP* Immunoprecipitation, *MALDI-TOF* Matrix-assisted laser desorption/ionization time-of-flight mass spectrometry, *MSD* Meso Scale Discovery, *NfL* Neurofilament light chain, *PET* Positron emission tomography, *p-tau* Phosphorylated tau, *p-tau181/p-tau217/p-tau231/p-tau396* Tau phosphorylated at Thr181, Thr217, Thr231, or Ser396, respectively, *Simoa* Single Molecule Array, *WB* Western blotting

Complementing this work, Yu et al. (2011) performed a histopathological and immunohistochemical comparison between human AD brains and aged dogs with cognitive dysfunction. They reported that aged dogs develop abundant Aβ deposits in cortical and hippocampal regions, including parenchymal plaques and vascular Aβ in distribution reminiscent of human cerebral amyloid angiopathy (CAA). However, canine plaques were generally more diffuse and were not accompanied by the full spectrum of amyloid plaque and neurofibrillary tangle pathology characteristics of advanced human AD. These findings support CCD as a partial - but biologically informative - AD-like pathology, in which amyloid deposition is prominent while downstream tau aggregation is comparatively limited (Yu et al. 2011).

The biochemical complexity of canine Aβ deposits was further refined after demonstrations that pyroglutamate-modified Aβ (AβpN3) accumulates in an age-dependent manner and becomes a major component of amyloid plaques (Chambers et al. 2011), thus mirroring human AD, where pyroglutamate Aβ is considered particularly aggregation-prone and neurotoxic (Bayer 2022). Parallel work by Frost et al. (2013) examined pyroglutamate-3 Aβ across species and reported robust deposition in dogs, further reinforcing that canine plaques can contain post-translationally modified Aβ species associated with aggregation and toxicity in humans (Frost et al. 2013).

Breed-specific heterogeneity in Aβ deposition was characterized across dogs from different ages (Mesquita et al. 2021). While both parenchymal plaque formation and vascular Aβ deposition correlated positively with age, significant inter-individual variability persisted, driven by breed and body size. These deposits spanned multiple regions, specifically the frontal, temporal, and occipital cortices, where CAA frequently co-occurred with perivascular microhemorrhages, indicating Aβ-mediated vascular fragility. Although no direct correlation between plaque density and CAA was established, the dual manifestation of these deposits mirrors the neurodegenerative hallmarks observed in human pathology.

A key question in CCD is how closely brain Aβ burden tracks with cognitive decline. Ozawa et al. (2016) correlated amyloid plaques, CAA, ubiquitin-positive granules, astrocytosis, microgliosis, and neuronal counts with questionnaire-based CCD severity scores. They observed that plaque density increased until 14 years of age and then declined in the oldest dogs, whereas CAA continued to increase with age. Notably, CCD severity correlated more strongly with glial activation and markers of synaptic or myelin damage than with plaque or CAA severity, suggesting that amyloid pathology alone may be insufficient to explain clinical impairment (Ozawa et al. 2016).

More recently, Hines et al. (2023) evaluated gliosis, Aβ1–42 aggregation, and tau hyperphosphorylation in aged dogs with and without CCD. Aggregated Aβ1–42 increased in cortical and hippocampal regions compared with young controls, confirming Aβ accumulation as a robust feature of canine brain aging. Interestingly, Aβ burden was similar in CCD-positive and age-matched CCD-negative dogs, whereas CCD dogs uniquely exhibited p-tau217 immunoreactivity, supporting the view that Aβ deposition may be necessary but not sufficient for overt cognitive decline and that downstream tau and neuroinflammatory processes likely modulate clinical conversion (Hines et al. 2023).

Taken together, available data indicate that the relationship between brain Aβ deposition and CCD severity is complex. Some dogs exhibit substantial amyloid pathology without marked cognitive impairment, whereas others show CCD despite similar Aβ burdens, potentially reflecting differences in tau pathology, neuroinflammation, synaptic vulnerability, or vascular involvement. These features position canine aging as a valuable platform for studying early and intermediate phases of amyloid dysregulation and its interaction with vascular and glial responses, while highlighting species-specific nuances that should be considered when extrapolating to human AD.

In CSF, Aβ dynamics do not follow a simple monotonic trajectory but instead suggest a stage-dependent sequence of molecular changes. One of the most compelling demonstrations of this non-linear pattern comes from Stylianaki et al. (2020), who reported elevated CSF Aβ42 in mildly cognitively impaired dogs, followed by a decline in severely impaired individuals (Stylianaki et al. 2020). Dogs in the early symptomatic phase showed higher Aβ42 concentrations than both cognitively unimpaired older dogs and those with advanced CCD, forming an inverted-U pattern. This profile resembles the proposed early compensatory phases in human biomarker trajectories, in which increased soluble Aβ production may precede sequestration into insoluble aggregates (de Leon et al. 2018; Gaubert et al. 2019). Similar early increases in soluble Aβ species before extensive plaque deposition have also been documented in beagle models during the presymptomatic phase of amyloidosis (Head et al. 2010).

Importantly, dogs exhibit a human-like repertoire of CSF Aβ isoforms. Using immunoprecipitation followed by MALDI-TOF/TOF mass spectrometry, Sarasa et al. (2013) identified multiple Aβ species ranging from Aβ33 to Aβ42, including several N-terminally truncated peptides implicated in early AD pathology (Sarasa et al. 2013). This biochemical overlap supports conserved APP processing and Aβ aggregation pathways across species, strengthening the rationale for translational comparisons.

Further supporting this interpretation, Alsulami et al. (2025) showed that CSF Aβ42 levels decrease with age and inversely correlate with cortical amyloid burden assessed *post-mortem*. In addition, the CSF Aβ1–42/Aβ1–40 ratio was reduced in CCD-positive dogs and correlated with CADES scores, consistent with the concept that ratio-based measures may better reflect disease-relevant amyloid shifts than absolute peptide concentrations (Alsulami et al. 2025). Regional data also suggest that Aβ1–42 aggregates preferentially in prefrontal and hippocampal regions, which are closely linked to cognitive decline (Hines et al. 2023). Correspondingly, aging dogs with cortical Aβ1–42 accumulation have been shown to exhibit reduced CSF Aβ42 levels once plaque load exceeds a threshold (Head et al. 2010).

Notably, the non-monotonic CSF Aβ pattern observed in multiple canine studies aligns with mounting evidence from human cohorts showing that CSF Aβ concentrations do not decline immediately. Still, it may transiently rise or fluctuate as APP cleavage shifts toward increased soluble Aβ production (Jack et al. 2009). In this sense, dogs may partially recapitulate the early, dynamic phases of amyloid dysregulation observed in human AD progression.

Several factors likely contribute to the variability in CSF Aβ trajectories observed across canine studies, including breed, body size, comorbidities, and methodological differences in sample handling or assay platforms. Nonetheless, across multiple cohorts, the pattern that emerges is consistent with a biologically meaningful, stage-specific shift from early elevations in soluble Aβ42, as brain amyloidosis begins, to later reductions as cortical Aβ accumulates, and soluble pools diminish.

Given the growing clinical impact of blood-based biomarkers (BBMs) in Alzheimer’s disease research (Hansson et al. 2022), blood-derived analytes represent an attractive and scalable approach for CCD detection and monitoring in veterinary practice. As in human AD, extracellular Aβ40 and Aβ42 are central to amyloid plaque formation and cerebral amyloid angiopathy in aging dogs, processes associated with cognitive deficits (Ozawa et al. 2016; Papaioannou et al. 2001).

One of the earliest studies evaluating plasma Aβ reported that younger and middle-aged dogs with minimal or undetectable amyloid pathology exhibit the highest circulating Aβ42 concentrations, which progressively decline as cognitive impairment becomes more severe (Gonzalez-Martinez et al. 2011). A subsequent longitudinal study found that Aβ42 concentrations were significantly higher in dogs with CCD compared with mildly impaired and cognitive unimpaired groups, whereas systemic inflammatory marker levels did not differ across groups (Schutt et al. 2015). More recent studies further suggest that mild cognitive impairment in dogs may be characterized by intermediate - or even transiently elevated - Aβ42 concentrations and Aβ42/Aβ40 ratios, followed by reductions in advanced CCD, consistent with progressive sequestration of soluble Aβ into insoluble brain deposits (Alsulami et al. 2025).

These investigations have refined the relationship between aging, cognitive status, and plasma Aβ dynamics. Panek et al. 2021) reported that Aβ40 and Aβ42 increase with age, accompanied by a progressive reduction in the Aβ42/Aβ40 ratio in plasma and by worsening cognitive performance, reinforcing the notion that ratio-based measures may outperform absolute peptide concentrations as indicators of clinically meaningful change (Panek et al. 2020). Other studies similarly reported elevated plasma Aβ42 levels in dogs diagnosed with CCD compared with healthy controls (Schutt et al. 2016). However, inconsistencies across cohorts are particularly evident when relying solely on total Aβ quantification, likely reflecting biological variability and methodological differences in assay platforms and pre-analytical handling.

Notably, the most comprehensive fractionation study to date demonstrated that plasma-bound and total Aβ42 fractions, rather than free peptide levels alone, best differentiate mildly impaired from unimpaired older dogs (Stylianaki et al. 2020). These biomarker alterations correlated with CSF Aβ42 and cognitive scale scores, suggesting preserved central-peripheral coupling in early disease stages and highlighting the potential utility of multi-analyte plasma panels that incorporate free, bound, and total Aβ42/Aβ40 measures to capture stage-dependent amyloid dynamics.

Critically, several sources of variability likely contribute to inconsistencies in Aβ findings across studies. First, breed composition and body size vary substantially across cohorts: large-breed dogs tend to have lower Aβ burdens than small breeds, which may confound cross-study comparisons. Second, assay platform heterogeneity, including differences between ELISA-based methods, SIMOA, and mass spectrometry, affects both absolute concentrations and inter-laboratory reproducibility. Third, cognitive phenotyping instruments differ across studies (e.g., CADES vs. CCDR vs. cognitive performance tasks), leading to variation in how cognitive groups are defined and potentially grouping animals at different biological stages under the same clinical label. Fourth, pre-analytical factors, including CSF and plasma storage conditions, freeze-thaw cycles, and collection timing, may introduce variability in Aβ measurements independent of true biological differences. These methodological heterogeneities, rather than true biological inconsistency, likely explain much of the discordance in published Aβ data and underscore the need for harmonized protocols before definitive conclusions about Aβ diagnostic utility can be drawn.

Overall, converging evidence indicates that Aβ dysregulation is a key pathological feature of CCD, but plasma (and CSF) Aβ measures do not yet provide sufficient sensitivity and specificity for a standalone clinical diagnosis or disease monitoring. Translation into routine clinical application will require longitudinal sampling across defined cognitive stages, improved harmonization of assay platforms, and integration with complementary biomarkers described in subsequent sections, particularly NfL, tau-related measures, and GFAP. An amyloid/tau/neurodegeneration [AT(N)]-like framework adapted to veterinary neuroscience may help contextualize blood Aβ results within broader pathophysiological staging, thereby improving diagnostic precision and translational interpretability.

### Tau

Tau is located in the axons, dendrites, nucleus, cell membrane, and synapses of neurons. The protein is also expressed to a lesser extent in astrocytes and oligodendrocytes. The main function of tau is to promote the assembly and stabilization of microtubules in neuronal axons (Imbimbo et al. 2023). Tau also plays a role in several other biological processes, including myelination, neurogenesis, motor function, learning, and memory (Kent et al. 2020).

While Aβ pathology is extensively characterized in aged dogs, the usefulness of tau as a biomarker for CCD remains elusive. Current neuropathological evidence suggests that aged and cognitively impaired dogs exhibit tau hyperphosphorylation, mislocalization, and synaptic accumulation. These features are more closely aligned with early-stage tauopathy in dogs than with the mature neurofibrillary tangles (NFTs) characteristic of advanced neurodegeneration (Abey et al. 2021). So far, NFTs have been identified only rarely in the canine brain; for instance, in one dog in one study (Smolek et al. 2016) and in three dogs in another study (Schmidt et al. 2015).

Multiple phosphorylation sites have been investigated in canine brain tissue. Hines et al. (2023) reported increased p-tau181 and p-tau217 immunoreactivity in cortical regions of aging dogs, with p-tau217 detected preferentially in dogs with CCD, suggesting potential disease relevance of this epitope (Hines et al. 2023). In addition, significant increases in specific p-tau isoforms (p-tau205 and p-tau214) were observed in synaptosomal fractions from CCD-affected dogs compared with controls, indicating synaptic tau pathology associated with cognitive decline (Smolek et al.  2016). At the synaptic level, increased phosphorylated tau isoforms, including p-tau205 and p-tau214, were observed in synaptosomal fractions from CCD-affected dogs compared with controls, indicating that tau pathology in CCD may be enriched at synapses and linked to cognitive decline (Smolek et al. 2016). In addition, widespread p-tau396 immunoreactivity across regions involved in learning and memory was reported, including the hippocampus, thalamus, and frontal cortex, whereas neurofibrillary tangle-like structures were rare, consistent with an early tauopathy phenotype (Abey et al. 2021). Further reports indicate intracellular co-localization of Aβ oligomers with phosphorylated tau deposits in hippocampal and cortical neurons in CCD, supporting a pathogenic interplay between oligomeric Aβ and tau dysregulation (Habiba et al. 2021).

In human AD, elevated CSF total tau (t-tau) and phosphorylated tau (p-tau) strongly reflect neuronal injury and correlate with disease severity (Teunissen et al. 2025). In dogs, however, available evidence suggests a more subtle and heterogeneous tau pathology (Vikartovska et al. 2021), limiting the direct translation of CSF tau biomarkers into clinically useful tools for CCD.

Despite this biological substrate, CSF-based studies have not yet demonstrated consistent or robust changes in tau concentrations associated with CCD. The limited available reports suggest that t-tau and p-tau levels in CSF are either unchanged or only minimally elevated in cognitively impaired dogs compared with age-matched controls, failing to show diagnostic utility under current analytical conditions *(*Vikartovska et al. 2021; Alsulami et al. 2025).

This inconsistency is likely to reflect both biological and methodological factors, particularly the disconnect between tissue-level tau accumulation - predominantly pre-tangle and synaptic - and the limited tau release into extracellular fluid. Unlike humans, who develop abundant neurofibrillary tangles as disease progresses, dogs typically exhibit pre-tangle and synapse-centric tau pathology, which may result in lower extracellular tau release into CSF (Abey et al. 2021). In addition, commercially available immunoassays used to measure tau were designed and validated for human tau isoforms, potentially reducing sensitivity in dogs due to minor sequence and epitope differences. Small sample sizes, heterogeneous clinical staging methods, and variability in CSF collection procedures further hinder reliable measurement.

Recent translational efforts have begun to quantify tau (total or phosphorylated) in CSF and plasma of aged dogs with cognitive impairment (Alsulami et al. 2025; Yoon et al. 2025). However, consistent diagnostic-grade elevations in CSF tau have not been established, reinforcing that tau currently remains an exploratory biomarker in CCD.

Nonetheless, tau remains a conceptually important marker because it reflects downstream neuronal damage not captured by amyloid biomarkers. As more sensitive assays tailored to canine tau are developed and larger longitudinal cohorts with neuropathological validation become available, CSF tau may eventually contribute meaningfully to biomarker panels for monitoring disease progression. For now, CSF tau should be viewed as an emerging, still-experimental indicator of neurodegeneration in CCD, whose future translational value depends on technical advancements and continued rigorous investigation.

Tau in blood has attracted considerable interest as a biomarker of neurodegeneration in human AD. Plasma phosphorylated tau species, particularly p-tau181 and p-tau217, show strong diagnostic and prognostic performance across the AD continuum (Janelidze et al. 2025). In dogs, however, available evidence remains limited, and tau measured in blood has not yet emerged as a useful biomarker for CCD (Vikartovska et al. 2021).

Key studies assessing peripheral tau in canine aging and cognitive decline are summarized in Table 1. To date, the only study that has systematically evaluated tau in blood in the context of early cognitive decline is the work by Vikartovska and colleagues (2021), who used ultrasensitive SIMOA assays to quantify serum tau, NfL, and Aβ42 in pet dogs classified as mildly cognitively impaired, normally aging older dogs, or young controls. They reported a clear increase in serum NfL in mildly impaired dogs compared with both aging and young controls, whereas serum tau concentrations did not differ across groups, with values remaining in a narrow range (Vikartovska et al. 2021). Importantly, the absence of a peripheral tau signal was consistent with neuropathological observations in the same cohort, in which AT8 (p-tau202/205)-positive neurofibrillary tangle-like pathology was not detected, despite abundant Aβ plaques and microglial activation (Vikartovska et al. 2021).

Accordingly, subsequent blood-based biomarker research in CCD has pivoted away from tau, focusing instead on analytes with more robust and reproducible associations with cognitive decline. NfL and, to a lesser extent, GFAP, an indicator of astrogliosis, have emerged as more reliable correlates of clinical impairment (Alsulami et al. 2025; Yoon et al. 2025). Machine-learning approaches have also prioritized metabolic and inflammatory proteins, including candidates such as RBP4, CXCL10, and NOX4, while tau has not been retained as a core feature in predictive models (Kim et al. 2024). This pattern suggests that, under current experimental conditions, peripheral tau provides limited incremental value relative to more informative blood biomarkers in CCD.

Several biological and analytical factors may explain these results. Neuropathological data indicate that canine tau pathology is typically subtle, diffuse, and enriched in pre-tangle or synaptic compartments, with hyperphosphorylated tau accumulating in synapses and mislocalized within neurons, whereas classical neurofibrillary tangles are rare in most CCD brains (Hines et al. 2023). Such a phenotype may generate only modest extracellular tau release and minimal spillover into the bloodstream, particularly in the absence of extensive neuronal loss.

The apparent disconnect between robust neuropathological evidence of tau hyperphosphorylation in canine brain tissue and the absence of consistent, diagnostically informative changes in CSF or blood tau warrants critical discussion. At least two interacting factors explain this discordance. Biologically, CCD tau pathology is predominantly pre-tangle and synapse-centric: hyperphosphorylated tau accumulates in synaptic compartments and is mislocalized within neurons, rather than forming the extracellular-accessible neurofibrillary tangles that drive tau release in human AD. This compartmentalization likely limits tau spillover into CSF and blood, resulting in minimal and inconsistent fluid-level changes even when substantial intraneuronal pathology is present. Technically, most available tau immunoassays were developed and validated for human tau isoforms and epitopes (e.g., AT8, AT100, pSer181, pThr217). Their cross-reactivity with canine tau may be incomplete, and quantitative accuracy may be compromised by epitope differences that have not been systematically characterized. Together, these biological and technical factors provide a coherent explanation for why robust tissue-level tau pathology has not translated into clinically useful fluid biomarker signals under current conditions, and underscore that the absence of a CSF tau signal in CCD should not be interpreted as the absence of tau pathology.

From a translational standpoint, this does not mean that tau in blood should be abandoned altogether, but it does require reframing expectations. In contrast to humans, in which plasma p-tau217 and p-tau181 are now leading blood biomarkers for AD pathology and are beginning to be used for trial screening and risk stratification (Ossenkoppele et al. 2025), no published study has yet reported plasma phosphorylated tau species in CCD dogs, and the only available data on total tau in blood are negative. Any future progress will likely depend on the development of canine-optimized assays targeting specific p-tau epitopes (such as p-tau181 or p-tau217), careful longitudinal design spanning preclinical to advanced CCD, and integration of blood tau with complementary markers (Aβ, NfL, GFAP, metabolic and inflammatory proteins) and imaging. Until such data emerges, tau in blood should be presented as an interesting but still speculative avenue, rather than as a practical biomarker in the current CCD biomarker toolkit.

### Neurofilament light chain (NfL)

NfL is a major cytoskeletal component of large-caliber, myelinated axons, and its release into cerebrospinal fluid CSF and blood is widely recognized as a sensitive marker of neuroaxonal injury (Khalil et al. 2024). In humans, neurodegenerative diseases, including Alzheimer’s disease, CSF and plasma NfL concentrations increase with normal aging and show pronounced elevations in conditions associated with axonal degeneration, making NfL one of the most robust biomarkers of neurodegeneration currently available (Khalil et al. 2018).

The main NfL studies are summarized in Table 1. In dogs, no study has yet directly quantified NfL protein levels in brain tissue from animals with CCD. Still, neuropathological and neuroanatomical data strongly support the presence of axonal pathology in regions relevant to cognition. Classic immunohistochemical work in dogs showed that neurofilament-positive neurons are particularly abundant among medium- and large-sized pyramidal cells in layers III and V of the neocortex, indicating that long-range corticocortical and corticofugal projections are particularly rich in neurofilament proteins and therefore primary sources of NfL release when axons degenerate (Hof et al. 1996).

Subsequent ageing studies in dogs have documented a range of degenerative changes in brain tissue, including neuronal loss, gliosis, and axonal and white-matter degeneration. In a seminal morphologic study of 20 old versus 10 young dogs, Borràs et al. (1999) reported age-associated changes involving meninges, choroid plexus, vessels, neurons, and glial cells, using immunohistochemistry for glial fibrillary acidic protein, neurofilament, ubiquitin, and β-amyloid (Borras et al. 1999).

Evidence from veterinary neurology first demonstrated that CSF NfL concentrations are substantially elevated in dogs with acute or chronic CNS injury, including meningoencephalitis of unknown origin and intracranial tumors, confirming the strong biological coupling between axonal degeneration and CSF NfL release (Yun et al. 2023). The relevance of NfL for aging and cognitive decline emerged more recently. In a comprehensive biomarker study of pet dogs spanning young, adult, and senior age groups, with and without cognitive impairment, CSF NfL levels increased robustly with age and were significantly higher in dogs with cognitive impairment compared with age-matched controls (Alsulami et al. 2025). Importantly, CSF NfL correlated not only with chronological age but also with owner-reported and clinician-evaluated measures of cognitive dysfunction, indicating that NfL reflects disease-relevant neurodegeneration rather than merely senescence (Alsulami et al. 2025).

These results align with parallel findings in plasma from cognitively impaired dogs, where NfL shows strong associations with age and impairment severity (Vikartovska et al. 2021). Although plasma NfL has received greater attention due to its clinical feasibility, CSF NfL remains a more mechanistically relevant measure of central axonal damage. Also, it provides crucial validation of peripheral NfL as a surrogate for CCD-related neurodegeneration. Together, the CSF and plasma data suggest that NfL may serve as a staging biomarker across the canine aging spectrum, mirroring its role in human Alzheimer’s disease and reinforcing the translational value of the canine model.

Notably, NfL stands out within the CCD biomarker landscape. Unlike tau, which shows subtle and inconsistent fluid-level changes due to the predominantly pre-tangle tauopathy in dogs, NfL reliably reflects the axonal injury and synaptic degeneration thought to underlie cognitive decline (Vikartovska et al. 2021; Alsulami et al. 2025).

The introduction of ultrasensitive SIMOA technology represented a major advance for canine biomarker research by enabling reliable quantification of NfL in blood. In the first translational study, mean NfL concentrations rose from approximately 5 pg/mL in puppies and juveniles to ~ 47 pg/mL in senior and geriatric dogs, reaching ~ 80–100 pg/mL in animals diagnosed with degenerative myelopathy or CCD (Panek et al. 2020). These age-related trajectories closely parallel those reported in humans, supporting the cross-species validity of NfL as a marker of neuroaxonal injury. Subsequent studies strengthened the association between blood NfL and cognitive status. In a population of aging pet dogs, Fefer et al. (2022) integrated behavioral questionnaires (CADES, CCDR), cognitive-performance tasks, and plasma NfL measurements, showing that higher NfL levels correlated with poorer inhibitory control (r² ≈ 0.48) and higher CADES scores (Fefer et al. 2022). These results provided the first direct evidence that plasma NfL reflects executive dysfunction and may help identify dogs in the early stages of cognitive decline.

Taken together, available evidence indicates that CSF NfL represents a biologically grounded and clinically promising biomarker of neuroaxonal injury in aging dogs. As longitudinal cohorts with standardized cognitive phenotyping become available, CSF NfL and its plasma counterpart are well-positioned to serve as central components of multimodal biomarker frameworks for characterizing CCD trajectories and monitoring disease progression, thereby strengthening the translational relevance of the canine model for human neurodegenerative research.

Additional support comes from studies using immunomagnetic reduction (IMR) technology, which show that dogs with CCD exhibit significantly higher plasma NfL concentrations than cognitively normal controls, reinforcing the diagnostic potential of peripheral NfL for early detection of CCD (Yoon et al. 2025).

More recently, a comprehensive biomarker study incorporating paired plasma and CSF samples revealed parallel increases in NfL concentrations in both compartments with advancing age and cognitive decline (Alsulami et al. 2025). Dogs classified as CCD-positive showed significantly higher plasma and CSF NfL, and plasma concentrations correlated strongly with owner-reported impairment (CADES, CCDR) as well as with CSF NfL, confirming that peripheral NfL reflects central axonal pathology (Alsulami et al. 2025). These findings align with emerging studies that emphasize that elevated NfL is among the most consistent and reproducible BBM alterations in CCD, alongside age-related changes in Aβ peptides (Yoon et al. 2025).

Detailed analyses of breed-specific cohorts have also refined our understanding of physiological modifiers of pNfL. In a study of healthy Labrador Retrievers, plasma NfL showed a moderately strong positive correlation with age (Spearman’s ρ = 0.68, *p* < 0.0001). In contrast, height and weight showed weak-to-moderate negative correlations with NfL. A bivariate regression model indicated that both increasing age and decreasing weight independently predicted higher NfL concentrations (Perino et al. 2021). This pattern partially contrasts with that of Panek and co-workers (2020), who found no effect of body weight on NfL levels in a more heterogeneous population. These discrepancies likely reflect differences in breed, morphology, and population structure, as well as the narrower weight range typical of single-breed studies.

For a truly translational framework, future studies should combine in vivo NfL measurements with post-mortem NfL immunohistochemistry or ELISA in well-characterized CCD and control brains to map regional axonal loss and correlate it with cognitive stage and other biomarkers (such as Aβ, tau, GFAP). Such work is essential to move from the current, largely inferential model, where fluid NfL is assumed to reflect brain axonal degeneration, to a validated neuropathological anchor for NfL as a biomarker of CCD. This would help to establish plasma NfL as a robust, minimally invasive biomarker of neurodegeneration in aging dogs. Nevertheless, although NfL is not disease-specific, its sensitivity to axonal injury, strong age-association, and consistent elevation in cognitively impaired dogs position it as a valuable tool for staging neurodegeneration, monitoring progression, and potentially evaluating therapeutic response in naturally occurring canine cognitive decline.

From a clinical standpoint, NfL’s most immediate utility in veterinary practice lies in disease staging and longitudinal monitoring rather than in differential diagnosis. Given its non-specificity across neurological conditions, a single elevated NfL value cannot distinguish CCD from other causes of neuroaxonal injury (e.g., meningoencephalitis, intracranial neoplasia, degenerative myelopathy). However, serial NfL measurements in dogs already diagnosed with CCD or at high risk due to age and breed profile can provide quantitative information on neurodegeneration rate - analogous to the pharmacodynamic role that NfL currently serves in human AD trials, where it tracks target engagement and neuroprotective effects of candidate therapies (Khalil et al. 2024). This pharmacodynamic application positions NfL as a particularly attractive primary or secondary outcome measure for future interventional studies in dogs, including trials of neuroprotective diets, environmental enrichment protocols, or disease-modifying drugs. The strong concordance between plasma and CSF NfL across studies (Alsulami et al. 2025) is especially relevant here: it confirms that minimally invasive blood-based sampling can substitute for CSF collection in longitudinal clinical monitoring, substantially improving feasibility and animal welfare in both clinical and research settings.

A critical prerequisite for translating NfL into routine veterinary clinical use is the establishment of normative reference ranges stratified by breed, body size, and age. Available data indicate that plasma NfL is influenced by both age (positively) and body weight (negatively), and that single-breed cohorts yield narrower and more informative reference intervals than mixed-breed populations (Perino et al. 2021). The development of breed-specific and size-adjusted thresholds - similar to breed-specific cardiac and hematological reference ranges already used in veterinary practice - would allow individual dogs to be compared against normative expectations rather than population means, enabling earlier and more sensitive detection of aberrant NfL trajectories. In parallel, the field would benefit from prospective longitudinal studies that track NfL from cognitively normal aging through the onset and progression of CCD, generating trajectory data analogous to those produced by the Alzheimer’s Disease Neuroimaging Initiative (ADNI) in humans. Such datasets would define inflection points and rates of change that are clinically meaningful - for example, identifying the NfL trajectory slope that best predicts conversion from normal aging to mild CCD within a defined time window - and would provide the evidentiary foundation needed to implement NfL as a validated staging and monitoring tool in veterinary neurology.

Taken together, these data indicate that axons in the canine brain (and to a lesser extent, spinal cord) are the most plausible source of elevated NfL in the blood and CSF of dogs with CCD. Data also highlights an important gap: brain tissue NfL has not yet been directly quantified as a CCD biomarker. While its high sensitivity across diverse neuropathologies inherently limits its diagnostic specificity, this pan-neuronal nature underscores its value as a robust progression marker. Consequently, NfL holds significant promise as a pharmacodynamic surrogate in longitudinal studies and future interventional trials targeting canine neurodegeneration.

### Glial fibrillary acidic protein (GFAP)

GFAP is an intermediate filament protein expressed predominantly by astrocytes and is widely used as a marker of astroglial activation and gliosis (Ozcelikay-Akyildiz et al. 2025). In human Alzheimer’s disease and related neurodegenerative conditions, GFAP has emerged as a sensitive indicator of astrocytic reactivity and disease progression, including in preclinical and genetically at-risk populations (Marksteiner and Humpel 2025; Ozcelikay-Akyildiz et al. 2025).

In dogs, however, most of the robust GFAP data come from neurological disorders other than CCD. Early studies demonstrated that serum or CSF GFAP is markedly elevated in dogs with severe astrocytic injury, such as necrotizing meningoencephalitis and progressive myelomalacia, supporting GFAP as a reliable marker of acute or inflammatory astroglial damage in the canine central nervous system (Sato et al. 2013).

By contrast, evidence supporting GFAP as a biomarker of CCD is limited and less consistent. In one of the largest blood-based biomarker studies to date, Yoon et al. (2025) measured plasma Aβ40, Aβ42, GFAP, and serum NfL across dogs ranging from cognitively normal to clinically diagnosed CCD. While serum NfL showed robust, stage-dependent increases and strong diagnostic performance, plasma GFAP did not consistently discriminate between cognitive stages and did not substantially improve diagnostic accuracy when added to NfL-based models (Yoon et al. 2025).

Similarly, Alsulami et al. (2025) evaluated paired plasma and CSF biomarkers in aged dogs with and without cognitive impairment, including Aβ peptides, NfL, and GFAP. Although GFAP was included as part of a multimarker panel, the principal and most reproducible associations with cognitive decline were observed for reduced Aβ42/Aβ40 ratios and elevated NfL, whereas GFAP showed weaker and less consistent relationships with cognitive status (Alsulami et al. 2025).

Taken together, these findings suggest that, under current assay conditions and cohort sizes, GFAP in blood or CSF does not provide the same diagnostic or staging value as NfL in CCD. This does not negate the biological relevance of astrocytic activation in canine brain aging but rather indicates that GFAP may be more sensitive to overt inflammatory or necrotic processes than to the more subtle astroglial changes associated with naturally occurring cognitive decline.

Additional limitations further constrain the standalone utility of GFAP. GFAP is inherently non-specific and may be elevated in a wide range of CNS conditions, including encephalitis, spinal cord injury, neoplasia, and traumatic brain injury. As a result, elevated GFAP levels in aging dogs may reflect comorbid neurological pathology rather than CCD per se, complicating clinical interpretation in real-world veterinary settings (Ozawa et al. 2016).

Nevertheless, GFAP remains a conceptually important component of CCD biomarker research. Future studies employing highly sensitive, canine-optimized GFAP assays, larger and better-phenotyped cohorts, and longitudinal designs may clarify whether subtle GFAP changes track with disease onset or progression. Conceptually, GFAP is most likely to contribute value as part of a multimodal biomarker panel, complementing markers of amyloid dysregulation (Aβ) and neuroaxonal injury (NfL) by capturing astroglial and neuroinflammatory components of CCD pathophysiology, rather than serving as a primary diagnostic biomarker on its own.

Table 1 integrates biomarker studies evaluating core AD-related pathways in dogs, including amyloid dysregulation, tau alterations, axonal injury, and astroglial activation. It organizes studies by year, detailing the cognitive phenotyping approaches, assay technologies, and analytes measured in CSF or blood. Together, these studies form the foundation for an emerging AT(N)-like biomarker framework in the canine model and illustrate the translational value of naturally occurring CCD for comparative neurodegeneration research.

### Future perspectives

A critical limitation of the current CCD biomarker literature is the degree to which methodological heterogeneity across studies impedes direct comparisons and definitive conclusions. This heterogeneity operates along three main axes. First, cohort design varies substantially: age ranges span from juvenile controls (< 1 year) to geriatric dogs (≥ 15 years), while breed composition ranges from single-breed controlled cohorts (e.g., exclusively Labrador Retrievers or Beagles) to highly mixed community-based samples. Body size is an important covariate for NfL and likely other analytes, yet few studies systematically control for it. Sample sizes range from fewer than 20 to over 100 dogs per group, affecting statistical power. Definitions of CCD severity and exclusion criteria for comorbidities (e.g., concurrent neurological disease, systemic illness) also differ, leading to between-study variation in case purity. Second, assay platform heterogeneity is substantial: conventional sandwich ELISA methods, which have picogram-per-milliliter sensitivity and are susceptible to matrix effects, are used alongside single-molecule array (SIMOA) technology capable of femtomolar detection; immunomagnetic reduction (IMR) platforms; and mass spectrometry approaches that enable isoform-level resolution. These platforms differ in sensitivity, dynamic range, coefficient of variation, and calibrator materials, making raw numerical comparisons across studies inherently unreliable without inter-platform harmonization studies. Third, cognitive phenotyping methods differ significantly: some studies rely on owner-completed questionnaires (CADES, CCDR), which capture caregiver-perceived functional decline but are susceptible to subjective bias; others use structured cognitive performance tasks (e.g., object permanence, reversal learning, inhibitory control), which are more objective but resource-intensive; and still others use clinician-assigned neurological status or exclude formal cognitive testing altogether. Since these instruments differ in the cognitive domains they assess and their sensitivity thresholds, animals at equivalent biological disease stages may be assigned to different cognitive groups across studies, contributing to apparent inconsistencies in biomarker-cognition correlations that may reflect classification noise rather than true biological variability.

Future research on CCD biomarkers should move decisively from proof-of-concept studies toward harmonized, longitudinal, and multicenter efforts capable of delivering clinically deployable tools. A critical priority is the standardization of diagnostic criteria, including harmonized thresholds for instruments such as CADES and CCDR, the incorporation of complementary cognitive tasks, and consistent clinical staging frameworks. In parallel, greater uniformity in sampling protocols - including CSF collection procedures, blood processing, storage conditions, and pre-analytical handling - is essential to reduce variability and improve cross-study comparability.

For amyloid biomarkers, longitudinal profiling of CSF and plasma Aβ42, Aβ40, and derived ratios across the full cognitive spectrum, from cognitively normal aging through mild impairment to advanced CCD, is essential to define temporal trajectories and inflection points analogous to the human AT(N) framework. Parallel neuropathological validation in subsets of dogs will be crucial to link fluid changes to regional plaque burden and vascular amyloid pathology. These efforts should be extended to a broader range of breeds and body sizes to reflect the heterogeneity of the pet dog population observed in clinical practice.

In the tau domain, future work should prioritize the development and validation of canine-optimized assays, particularly for phosphorylated tau epitopes (e.g., p-tau181, p-tau217) that have transformed human AD biomarker research. Even if CCD never develops dense neurofibrillary tangles to the same extent as human AD, more sensitive tools may still reveal subtle, stage-dependent CSF tau changes that inform on synaptic dysfunction and downstream neurodegeneration. Such studies should be tightly integrated with quantitative neuropathology and advanced imaging (e.g., MRI-based volumetry and diffusion metrics) to clarify the relationships among pre-tangle tauopathy, structural brain changes, and cognition.

For NfL, the immediate goal is to move from cross-sectional associations to prognostic and therapeutic applications. Longitudinal plasma and CSF NfL measurements, obtained at multiple time points, can determine whether NfL trajectories predict conversion from normal aging to mild impairment, or from mild to advanced CCD, and whether they respond to pharmacological or lifestyle interventions. Incorporating NfL into interventional and preventive trials in dogs will help establish its role as a pharmacodynamic biomarker reflecting target engagement and neuroprotection. Breed-specific reference ranges and studies addressing the influence of morphology, body weight, and comorbidities will be necessary to optimize clinical interpretation.

GFAP and other glial or inflammatory markers including cytokines, chemokines, and microglia-related proteins, warrant further but cautious exploration. Their greatest potential likely lies in multimarker panels rather than in isolation, complementing Aβ and NfL by capturing astroglial and neuroinflammatory components of CCD. Machine-learning approaches that integrate clinical data, cognitive scores, imaging, and multi-analyte biomarker signatures are particularly promising for building predictive models capable of early risk stratification and individualized prognosis.

Translating CCD biomarkers into clinical veterinary practice requires addressing both diagnostic utility and implementation constraints. Among the markers reviewed, NfL emerges as the most clinically actionable candidate. Its consistent elevation in cognitively impaired dogs, robust correlation with owner-reported and performance-based measures, and availability in plasma make it the most suitable biomarker for disease staging and longitudinal monitoring in clinical settings. Plasma Aβ42/Aβ40 ratios may offer additional value for early detection - particularly in the presymptomatic or mildly impaired phases - but their non-linear trajectory and sensitivity to pre-analytical variables limit their use as standalone diagnostic tools without longitudinal calibration and breed-specific reference values. Tau and GFAP should currently be regarded as research-grade rather than clinically deployable biomarkers, pending further validation in larger, well-phenotyped canine cohorts. From a practical standpoint, an important distinction exists between biomarkers suited for diagnostic screening (identification of at-risk or early-stage animals) and those suited for disease monitoring or therapeutic response evaluation (tracking progression or response to intervention): NfL, by virtue of its consistent age - and disease-dependent trajectory, holds promise for both applications, whereas Aβ ratios are more informative in earlier disease stages. Key barriers to clinical implementation include the high cost and limited accessibility of ultrasensitive platforms such as SIMOA in routine veterinary practice, the absence of validated species-specific assays for most analytes, and the current lack of established reference ranges stratified by breed, age, and body size. Addressing these barriers will be essential to bridge the gap between research findings and clinical utility.

From an analytical standpoint, recent CCD biomarker studies increasingly employ ultrasensitive platforms such as SIMOA and MSD, improving detection limits for blood-based biomarkers (Alsulami et al. 2025; Fefer et al. 2022; Perino et al. 2021; Vikartovska et al. 2021). However, despite the high degree of molecular homology between dogs and humans, most commercially available assays remain optimized for human proteins. Their application to canine samples, therefore, relies heavily on cross-species antibody reactivity and requires cautious interpretation, as epitope recognition, analytical performance, and quantitative accuracy may differ in the canine context. Without assay harmonization and species-specific validation, cross-study comparisons will remain challenging, and true biological effect sizes may be obscured by methodological noise.

In addition to analyzing biomarkers in the most well-known fluids, such as cerebrospinal fluid, plasma, and serum, new technologies are seeking to identify biomarkers in the tear film for various human diseases, including AD. Compounds such as Lyzosyme-C, Lipocalin-1, Lacritin, and even Aβ and p-tau have been described as potential new biomarkers for the disease. Although similar studies using tear film have not yet been published in dogs with CCD, these findings point to new possibilities for biomarkers. Their advantages include low invasiveness, ease of sample collection, and the potential to develop low-cost rapid tests, facilitating disease diagnosis (Fotovat-Ahmadi et al. 2025; Kastelan et al. 2023; Krol-Grzymala et al. 2022).

Finally, CCD biomarker research should be explicitly framed within a One Health and translational neuroscience perspective. Well-characterized, biomarker-enriched canine cohorts offer a unique opportunity to bridge the gap between experimental rodent models and human clinical trials, providing a naturally occurring platform in which to evaluate preventive and disease-modifying strategies under real-world conditions of aging, comorbidity, and environmental exposure. Aligning methodological standards and outcome measures with those used in human dementia research will maximize bidirectional translational value, improving both veterinary care for aging dogs and the design of human AD trials.

## Conclusions

CCD has emerged as one of the most compelling spontaneous models of human AD, recapitulating key neuropathological hallmarks, including Aβ deposition, tau hyperphosphorylation, synaptic loss, neuroinflammation, and progressive cognitive decline, within a real-world, naturally aging population.

Collectively, available data indicates that amyloid- and neurodegeneration-related markers in dogs follow trajectories that are broadly analogous to those seen in human AD, but with species-specific nuances that must be carefully considered in translational work.

Among fluid biomarkers, CSF and plasma Aβ42 (and Aβ42/Aβ40 ratios) show stage-dependent, often nonlinear changes, with early increases in mildly impaired dogs followed by reductions in more advanced CCD, mirroring preclinical “compensatory” phases described in human AD. However, substantial methodological variability and biological noise mean that Aβ alone does not yet provide sufficient diagnostic robustness. Tau pathology in CCD brains, dominated by pre-tangle, synaptic, and mislocalized p-tau rather than abundant neurofibrillary tangles, has not translated into consistent, clinically informative changes in CSF or blood tau, which currently remain exploratory markers rather than practical tools.

In contrast, NfL consistently stands out as a sensitive and reproducible indicator of neuroaxonal injury. CSF and plasma NfL concentrations increase with age, are elevated in dogs with cognitive impairment, and correlate with owner-reported and performance-based cognitive measures. These convergent findings position NfL as the leading candidate biomarker for staging neurodegeneration and monitoring progression in CCD. GFAP, while biologically plausible as a marker of astroglial activation, has so far shown weaker and less consistent associations with CCD severity, underscoring its current status as a promising but unvalidated biomarker.

Overall, the available evidence supports a multimodal paradigm in which CCD is best characterized by integrated panels that capture amyloid dysregulation (Aβ), neuroaxonal damage (NfL), and potentially astroglial responses (GFAP), rather than by any single marker in isolation. Such an approach not only improves diagnostic precision in dogs but also strengthens the translational relevance of CCD as a One Health model for human dementia research, bridging companion animal medicine and human neurology. Positioning CCD within a biomarker-informed, One Health framework offers a unique opportunity to bridge veterinary neurology and human dementia research.

## Data Availability

All data generated or analyzed during this study are included in this published article.

## Abbreviations

Aβ: Amyloid-β
AβpN3: Pyroglutamate-modified Aβ
AD: Alzheimer’s disease
APP: Amyloid Precursor Protein
BBM: Blood-Based Biomarker
CADES: Canine Dementia Scale
CAA: Cerebral Amyloid Angiopathy
CCD: Canine Cognitive Dysfunction
CCDR: Canine Cognitive Dysfunction Rating Scale
CNS: Central Nervous System
CSF: Cerebrospinal Fluid
DISHAA: Acronym for clinical signs (Disorientation, altered Social interaction, sleep-wake cycle disturbances, House soiling, changes in Activity levels, and Anxiety)
ELISA: Enzyme-Linked Immunosorbent Assay
GFAP: Glial Fibrillary Acidic Protein
IMR: Immunomagnetic Reduction
NfL: Neurofilament light chain
NFT: Neurofibrillary Tangles
p-tau: Phosphorylated tau
SIMOA: Single-molecule array
t-tau: Total tau
